# Lazy Lips: Hyperkalemia and Acute Tetraparesis—A Case Report from an Urban Emergency Department

**DOI:** 10.1155/2014/160396

**Published:** 2014-11-25

**Authors:** Christian T. Braun, David S. Srivastava, Bianca Maria Engelhardt, Gregor Lindner, Aristomenis K. Exadaktylos

**Affiliations:** ^1^Department of Emergency Medicine, Inselspital, University Hospital Bern, Freiburgstrasse, 3010 Bern, Switzerland; ^2^Department of Surgery, Inselspital, University Hospital Bern, Freiburgstrasse, 3010 Bern, Switzerland; ^3^Department of Respiratory and Critical Care Medicine, Otto Wagner Hospital, Baumgartner Höhe 1, 1140 Vienna, Austria

## Abstract

A 58-year-old male patient was admitted to our emergency department at a large university hospital due to acute onset of general weakness. It was reported that the patient was bradycardic at 30/min and felt an increasing weakness of the limbs. At admission to the emergency department, the patient was not feeling any discomfort and denied dyspnoea or pain. The primary examination of the nervous system showed the cerebral nerves II–XII intact, muscle strength of the lower extremities was 4/5, and a minimal sensory loss of the left hemisphere was found. In addition, the patient complained about lazy lips. During ongoing examinations, the patient developed again symptomatic bradycardia, accompanied by complete tetraplegia. The following blood test showed severe hyperkalemia probably induced by use of aldosterone antagonists as the cause of the patient's neurologic symptoms. Hyperkalemia is a rare but treatable cause of acute paralysis that requires immediate treatment. Late diagnosis can delay appropriate treatment leading to cardiac arrhythmias and arrest.

## 1. Introduction

Hyperkalemia is common in emergency department patients with a prevalence rate of about 9%. About 3/4 of cases of hyperkalemia in emergency department patients were described to be caused by either acute or chronic renal failure or medications linked to this condition [1]. Hyperkalemia often appears clinically asymptomatic, and most often the electrolyte disorder gets only symptomatic when hyperkalemia is severe. Clinical features range from mild to life-threatening manifestations such as weakness to malign cardiac arrhythmias. We present the rare case of a severe neurologic manifestation of profound hyperkalemia.

## 2. Case Presentation 

A 58-year-old patient was admitted to the Emergency Department of the Inselspital, University Hospital Bern, by the Swiss air ambulance service.

At arrival it was reported that the patient felt an increasing weakness of the limbs for 3 days. On the day of admission, the weakness had intensified to such a high level that the patient called an ambulance.

He had usually a treatment for high blood pressure with aldosterone antagonists.

When asymptomatic bradycardia of 30 beats/min was diagnosed by the ambulance team, they suspected an acute coronary syndrome and treated the patient with P2Y12 receptor inhibitors, heparin, and aspirin. During transport, epinephrine needed to be administered to stabilize the now instable blood pressure. At arrival in the emergency department, the patient was not feeling any discomfort and denied dyspnoea or pain; the blood pressure was 154/58 mmHg, the heart rate was mildly bradycardic with 54 beats/min, tachypnea with a rate of 20/min was present, oxygen saturation was 100% with high-flow oxygen, temperature was 36.5°C, and the patient showed 15/15 points on the Glasgow Coma Scale (GCS) examination.

In the physical examination on arrival he showed a normal cardiovascular and gastrointestinal system; the examination of respiratory system was normal except for slight tachypnea.

The nervous system showed the cerebral nerves II–XII intact, the muscle strength of the lower extremities was 4/5, and a minimal sensory loss of the left hemisphere was observed. In addition, the patient complained about lazy lips.

**Figure 1:**
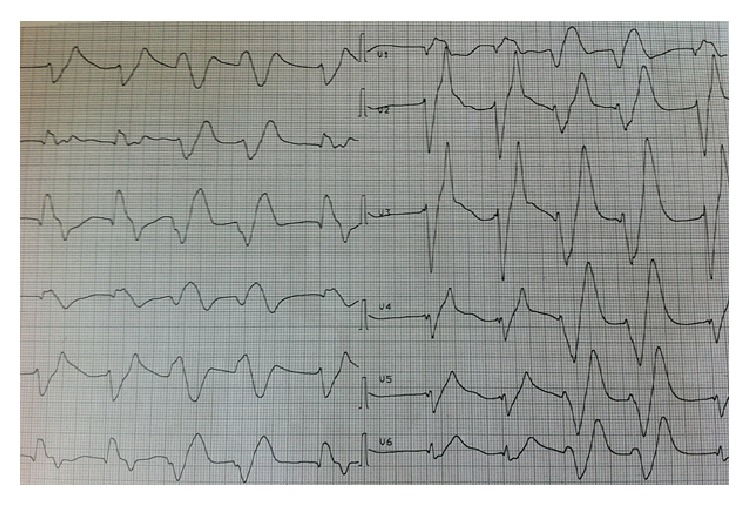
The electrocardiogram showing tall and peaked T waves, flattened and broadened P waves, and widened QRS complexes.

The electrocardiogram showed abnormalities, including tall and peaked T waves, flattened and broadened P waves, and widened QRS complexes (Figure 1). Because myocardial infarction could not be ruled out at that point, an emergency echocardiography study was conducted, which showed a normal result, especially normal left and right ejection fraction, no pericardial effusion, and a normal kinetic of the heart.

During this examination the patient developed a symptomatic bradycardia, accompanied by complete tetraplegia. The reevaluation of the neurologic system revealed a conscious and oriented patient with now areflexic paralysis of both lower and upper limbs.

Power in lower and upper limbs was 0 of 5; the Babinski sign was negative. There was hypoesthesia of the extremities, accentuated at the left side.

Continuously, the patient's vigilance declined to a GCS below 8, so that the patient was intubated for airway protection. Atropine was administered during the episode of bradycardia with low output and pending pulseless electric activity (PEA).

The laboratory analysis, which was available 1 hour after arrival of the patient in the emergency department, showed a serum potassium level of 9,9 mmol/L and sodium of 128 mmol/L, the chloride was 114 mmol/L, and the pH was 7,161 (pCO_2_ 25 mmHg, bicarbonate 10 mmol/L). Serum creatinine, without known chronic renal failure, was 167 *µ*mol/L with an estimated glomerular filtration rate of 38 mL/min. Fractional excretion of urea was 12%, which hinted towards prerenal failure.

After getting results on serum electrolytes,the patient was initially treated with calcium gluconate 10% and 8.4% sodium bicarbonate for membrane stabilization followed by 10 IE insulin within 50 mL glucose 40% given for 30 minutes for shifting of serum potassium into the cells and loop diuretics for renal potassium elimination [2, 3].

These therapeutic procedures stabilized the cardiac output and the general condition of the patient but were not therapeutic options for a longer period, so that the patient was transferred to the intensive care unit for hemodialysis for slow correction of hyperkalemia.

One day after correction of hyperkalemia, the patient was extubated and referred to the department of nephrology for further treatment.

**Figure 2:**
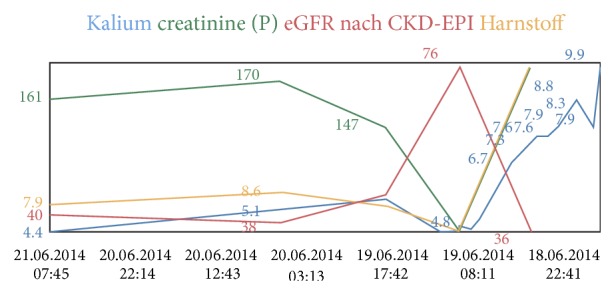
Trends of the laboratory parameters from admission to the emergency department until 2 days before discharge of the hospital: potassium, creatinine, GFR CKD-EPI, and urea.

During dialysis, the potassium and the arterial blood pH decreased to normal values, but the renal parameters stayed elevated (Figure 2). We assumed this to a prior existing renal insufficiency.

During the hospital stay, the antihypertensive therapy was changed to ACE-inhibitors and prior spironolactone therapy was stopped.

## 3. Discussion

Hyperkalemia often appears without any clinical symptoms, despite diagnostic signs like ECG changes including tall and peaked T waves, flattened and broadened P waves, and widened QRS complexes. Clinically, these patients often present with either bradycardia or tachycardia [2, 3]. Neurological symptoms like paresthesia/tetraparesis seem to appear only with extremely elevated potassium levels [4] and therefore appear quiet rarely [5, 6]. However, electrolyte disorders are common in emergency department patients and often cause unspecific symptoms. Thus, ordering an electrolyte panel or point of care testing should be standard in almost all patients presenting to an emergency department due to the potentially harmful effects of electrolyte disorders on patients outcome.

## References

[1] Pfortmüller C. A., Leichtle A. B., Fiedler G. M., Exadaktylos A. K., Lindner G. (2013). Hyperkalemia in the emergency department: etiology, symptoms and outcome of a life threatening electrolyte disorder. *European Journal of Internal Medicine*.

[2] Weisberg L. S. (2008). Management of severe hyperkalemia. *Critical Care Medicine*.

[3] Phiri T., Allain T. J., Dreyer G. (2013). Acute confusion and ataxia in the emergency department with an unexpected underlying diagnosis. *Malawi Medical Journal*.

[4] Panichpisal K., Gandhi S., Nugent K., Anziska Y. (2010). Acute quadriplegia from hyperkalemia: a case report and literature review. *Neurologist*.

[5] Delgado-Alvarado M., Palacio-Portilla E., Pelayo-Negro A. L., Lerena P., Berciano J. (2013). From ileostomy to sudden quadriplegia with electrocardiographic abnormalities: a short and unfortunate path. *Neurological Sciences*.

[6] Wahab A., Panwar R. B., Ola V., Alvi S. (2011). Acute onset quadriparesis with sine wave: a rare presentation. *The American Journal of Emergency Medicine*.

